# Comparison the effect of Otago and chair squat exercises on the fear of falling and the quality of life of the older adults, a clinical trial study

**DOI:** 10.1007/s40520-025-02951-7

**Published:** 2025-03-03

**Authors:** Zahra Mohammadi, Tayebeh Mirzaei, Ali Ravari, Zahra Kamiab

**Affiliations:** 1 Geriatric Care Nursing Master of Science Student, School of Nursing and Midwifery, Geriatric Care Research Center, Rafsanjan University of Medical Sciences, Rafsanjan, Iran; 2 Department of Medical-Surgical Nursing, School of Nursing and Midwifery, Geriatric Care Research Center, Rafsanjan University of Medical Sciences, Rafsanjan, Iran; 3 Department of Community Medicine, School of Medicine, Rafsanjan University of Medical Sciences, Rafsanjan, Iran

**Keywords:** Aged, Fear, Quality of life, Exercise

## Abstract

**Background:**

Staying at home limits older people's physical activity and increases their fear of falling. Also, their physiological and psychological problems lead to decrease in physical activity, which affects their quality of life.

**Aims:**

The aim of this study was to help use a cost-effective, and less complicated method to reduce the fear of falling and improve the quality of life of the aged people.

**Methods:**

A total of 126 aged people (over the age of 60) participated in this study, which lasted 8 weeks (three 45-min sessions per week at home). The participants were divided into three Chair squat, Otago, and control groups randomly. Fear of falling and quality of life scores were evaluated before and after the intervention. The data were analyzed using SPSS software.

**Results:**

Before the study, there was no significant difference between the groups in terms of fear of falling and quality of life. After the intervention, the Otago was more effective than chair squat exercise in reducing the average score of the fear of falling, but there was no significant difference between the intervention groups in terms of quality of life.

**Discussion:**

Both Otago and chair squat exercises were cost-effective and less complicated methods that helped reduce fear of falling and improve the quality of life of the older adults.

**Conclusion:**

Performing the Otago and chair squat exercises at home was effective in reducing fear of falling and improving quality of life of the older adults.

**Clinical trial registration:**

IRCT20150519022320N29 on July21, 2023.

## Introduction

From a historical perspective, the idea of an aging population is a relatively recent issue. It is noteworthy that in 1950, the proportion of the population over 65 was 11 percent in all countries. By 2000, the maximum percentage was 18 percent. By 2050, however, the issue will increase significantly to 38 percent. Also, in middle- and low-income Asia–Pacific countries, the percentage of the population 65 and older is predicted to rise by over 2.5 times during the next several decades, to reach 11% for men and 14.1% for women in 2050 [1, 2].

As people age, they spend more time at home due to physical reasons. Staying at home for a long time can reduce the amount of physical activity, mobility, and balance of the older adults and increase the risk of falling and, as a result, the fear of falling in them [3]. In addition, other physiological and psychological problems of the older adults, such as chronic pain, insomnia, anxiety and depression, can lead to a decrease in physical activities and, consequently, a decrease in their ability to perform daily activities, which affects their quality of life [4]. According to studies, 60 to 72.9 percent of the older adults are inactive [5].

Fear of falling (FOF) is a normal physical reaction that can have negative impacts on older adults. This reaction is presented as a potential health problem with the same level of importance as a fall. It is also described as fear of standing or walking. FOF subsequently refers to various concerns, such as a reduced ability to avoid falling during essential and non-hazardous activities of daily living and feeling insecurity during outdoor activities [6, 7]. The prevalence of FOF is different in each region, and in general, the amount of fear of falling among the older adults varies between 20.8 and 85% [6, 8]. High levels of fear of falling can cause reduced physical activity in the older adults [9].

Quality of life (QOL) refers to a wide range of subjective and multifaceted elements and concepts that are influenced by cultural and social contexts [6]. The QOL is classified by Lawton as appropriateness of behavior, psychological well-being, and an objective and observable environment. The QOL is defined by World Health Organization (WHO) as an abstract concept that can be improved, maintained, or diminished [10]. It also defines a person's perception of his/her position in life within the framework of the cultural and value system in which he/she lives and in relation to his/her goals, expectations, standards, and concerns [11].

In older adults at risk of falls; balance, functional ability, and muscle strength are associated with FOF and QOF [12]. Changes in the musculoskeletal system, especially in the upper and lower extremity and postural muscles, affects muscle strength and the ability to maintain balance, the body's ability to maintain its position at rest or during movement, and posture control in the older adults [13]. Older adults have low levels of physical fitness, which causes a gradual decrease in functional capacity, an increase in fat mass, a progressive decrease in muscle mass, a decrease in quality of life, and a loss of functional independence [14].

Exercise is considered the main method that improves the older adults physical condition, [15] and it is also useful as a first-line non-pharmacological treatment [16]. Doing exercise as complementary and non-pharmacological methods have a special application and place in nursing [17]. Furthermore, nursing holistic theories as well as nursing ethics confirm the necessity of using complementary treatments in nursing [18].

Exercise includes participating in planned, repeated, structured and purposeful physical activities to promote or maintain health or disease, which also improves balance, muscle strength and mobility [19, 20].

Among various Exercises, performing exercises at home helps the older adults to practice adaptation to movements and daily functional ability and increase their ability to perform life activities [21]. Home-based exercise is a part of exercise program designed in the home environment, which increases muscle strength and improves functional ability, and as a result it helps to reduce the risk of falling and increase the QOL of the older adults, [22] and can be used to maintain balance and restore physical function [23, 24].

One of the home-based exercises which is effective in preventing falls and improving the performance of the older adults by increasing the lower limbs strength, is the Otago Exercise Program (OEP) [21, 25]. OEP is a multidimensional exercise that combines warm-up, progressive muscle strengthening, balance, and aerobic exercises and walking sessions. Studies have shown that OEP can help improve the physical performance of older adults in the community through balance and strength exercises and reduce the occurrence of falls and fall-related injuries in them by 35% and also can enhance balance in older adults [15, 26].

Another balance and resistance exercise that can be performed at home for the older adults is the chair squat exercise (CSQ). This chair-based exercise helps strengthen the muscles needed for walking, going up and down stairs, and getting up from a chair [27]. The CSQ is a repeated transition from sitting to standing in a short period of time (10 sit-to-stand positions for 30 s, every 20 min) [28].

Both OEP and CSQ are balance- and muscle-strengthening exercises that can be performed by the older adults at home, but they are different in terms of energy consumption and complexity. OEP focuses on more physical factors than CSQ. Since extensive research has not been carried out on Otago and CSQ and the comparison of the effects of these two exercises on the FOF and the QOL of the older adults in this way, it was decided to compare the impact of Otago and CSQ exercise on the FOF and the QOL of the older adults in Rafsanjan.

## Materials and methods

The research design and the protocol used in the research were registered in the Iranian Registry of Clinical Trials (https://irct.behdasht.gov.ir/), with the identifier IRCT20150519022320N29.

### Participants

A total of 126 person over 60, were recruited in the study. After reviewing the electronic profiles of the older adults who referred to the comprehensive health service centers in Rafsanjan, Iran, the older adults who met the inclusion criteria informed completely about the study. Based on the study’s purpose and after filling out the questionnaires, participants signed informed consent and included in the study voluntarily. The inclusion criteria include age over 60 years, obtaining a minimum average score of 10 from the SAFFE questionnaire, having a low average QOL based on the SF-36 questionnaire, not having hearing, vision, physical, and mental problems, not suffering from diseases and taking medicines that cause imbalance and fear of falling, not living in a nursing home or other institutions, and not having an activity prohibition order from a doctor. The exclusion criteria include unwillingness to continue cooperation, not doing more than 3 sessions of exercises, and suffering from physical diseases that prevent full participation in training.

The total sample size based on the Raeisi Z et al. study [29], with a 15% possibility of people dropping out during the study, was calculated at 126 people (42 people in each group).

$$n_{2} = n_{3} = K \times n_{1} = \frac{{\left( {Z_{{1 - \frac{\alpha }{2}}} + Z_{1 - \beta } } \right)^{2} \times \left( {\sigma_{1}^{2} + \frac{{\sigma_{2}^{2} }}{k}} \right)}}{{\Delta^{2} }}$$

### Experimental design

This was a randomized controlled trial study. After obtaining the permission and submitting a letter of introduction from Iran, Rafsanjan University of Medical Sciences and receiving the ethic code from Iran, Rafsanjan research ethic committees (IR.RUMS.REC.1402.051), written consent obtained from the research units. 126 older adults were included in the study based on the inclusion criteria. The eligible participants completed a demographic profile questionnaire and were assured that their withdrawal from the study would not cause any issues for them. After establishing the sample framework, the participants were divided into three groups: A, B, and C. The initial allocation of participants to one of the three groups was conducted using a simple random method through random allocation software. The subsequent allocations for the remaining two groups were also performed in the same manner using the software. After equally distributing the participants among the three groups, intervention methods were written on three separate sheets, and one method was assigned to each group through a lottery system. The intervention groups consisted of the Otago exercise (n = 42), the CSQ (n = 42), and the control group (n = 42). Throughout the intervention, participants continued their regular medications and maintained their usual diet and lifestyle. During the study, two individuals were excluded: one from the Otago group and one from the control group.

After the mentioned assessments, the participants underwent pretests using the SAFFE and SF-36 questionnaires, so the average scores of FOF and QOL in the intervention and control groups were calculated and compared, respectively. After the completion of the intervention, the questionnaires were filled out again by the participants and were compared in three groups and also with the results of before the intervention.

### Training protocol

Before beginning the intervention, members of the intervention groups attended an in-person orientation session specific to their group with a member of their family which was conducted in the training hall of comprehensive health centers in Rafsanjan, Iran. The researcher taught the exercise protocol using booklets (which had pictures on one side and explanations on how to perform each movement on the other side) and educational videos (containing how to perform each movement correctly) with the aim of better understanding and in order to ensure the correct execution of the exercises. All participants done exercises that moment. At the end of the orientation session, all seniors were evaluated individually in terms of their correct performance of the exercises. They were also taught the principles of performing exercise and the necessary safety points during exercise in this meeting.

The Otago and CSQ intervention group members did the exercises related to their group based on Table 1, for three 45-min sessions per week at home individually according to the training approach, the booklets, and videos that they were provided. Every session started with warm-up (head, trunk, neck and ankle Movements and back Extension) and concluded with cool-down (Stretching the neck, trunk, and leg and stretching the back of the thigh) exercises. In the control group with the same conditions as the intervention groups, there was no intervention and they were told to continue their usual daily activities.

**Table 1:** The Otago and CSQ training schedule

Weeks	Exercises
The Otago training schedule	
First and second weeks	Warm-up, front knee strengthening, back knee strengthening, side hip strengthening, knee bends supported, heel toe stand supported, sit to stand using hands, stair walking, cool-down
Third and fourth weeks	Warm-up, front knee strengthening, back knee strengthening, side hip strengthening, knee bends no support, backwards walking supported, walk and turn, sideways walking supported, heel toe stand no support, one leg stand supported, sit to stand using one hand, stair walking, cool-down
Fifth and sixth weeks	Warm-up, front knee strengthening, back knee strengthening, side hip strengthening, calf raises supported, toe raises supported, knee bends no support, walk and turn, sideways walking no support, heel toe walking supported, one leg stand no support, heel walking supported, toe walking supported, sit to stand no hands, cool-down
Seventh and eighth weeks	Warm-up, front knee strengthening, back knee strengthening, side hip strengthening, calf raises no support, toe raises no support, knee bends no support, backwards walking no support, heel toe walking no support, one leg stand no support, heel walking no support, toe walking no support, heel toe walking backwards, sit to stand no hands, cool-down
The CSQ training schedule	
First to eighth weeks	Warm-up, 10 sit-to-stand positions for 30 s, repeat for 20 min, 5 min rest, 10 sit-to-stand positions for 30 s, repeat for 20 min again, cool-down

During the study, all the participants were monitored through virtual groups and telephone follow-ups to ensure proper and timely exercise, and they raised their questions and problems. Also, the exercises of each session were reminded and monitored by sharing videos of the exercises of each session.

### Measurements

#### Demographic information

Including age, gender, height, weight, education, marital status, number of falls, which were completed as self-reported by the older adults before the intervention.

All participants completed FOF and QOL questionnaires before (pre-values) and after the intervention (post-values).

#### FOF

To assess the FOF, we used the Survey of Activities and Fear of Falling in the Elderly (SAFFE) scale. SAFFE assessed FOF during the performance of 11 daily living activities, mobility tasks, and social activities. Based on the assumption that activity avoidance may be an early sign of fear of falling, the SAFFE measured information about participation in exercise activities and social activities. The SAFFE had 11 activity items, and each item had four options (0 = not at all worried, 1 = a little worried, 2 = somewhat worried, and 3 = very worried). The scoring range was 0–33 [30, 31]. SAFFE score > 10 meant a higher level of FOF and SAFFE score < 10 meant a lower level of FOF based on previous research [31]. Participants who had a FAFFE score > 10 were considered to have FOF. Its validity and reliability have been confirmed in Iran by Zarei et al. The validity ratio is 0.78 and Cronbach's alpha is 0.93 [32].

#### QOL

To assess the QOL, we used the SF-36 questionnaire. This questionnaire contains 36 questions that identify positive and negative states, focusing on functional status and emotional health [33]. The SF-36 evaluated eight subscales: well-being (five six-choice items), physical health (five five-choice items), physical function (ten three-choice items), role dysfunction due to physical health (four two-choice items), pain (two items, five- and six-choice, respectively), social function (two five-choice items), role dysfunction due to emotional health (three two-choice items), and energy fatigue (four six-choice items). The items were coded in a way that higher scores indicated better QOL [34]. Also, in Iran its validity and reliability have been verified by Jafari and colleagues, and Cronbach's alpha coefficient is 0.91 [35].

### Statistical analysis

The collected data underwent statistical analysis by SPSS software version 24 (IBM SPSS Inc., Chicago, IL, USA). Quantitative data results were reported as "standard deviation ± mean," while qualitative data were presented as "number (percentage)." To compare the means of quantitative variables among the three examined groups (Squat, Otago, control), one-way analysis of variance (ANOVA) was employed, and in case of significance, Post-hoc (Tukey’s multiple comparisons) test was used to compare the means of pairwise groups. Additionally, to compare the frequency distribution of qualitative variables in the three study groups, the chi-square test or Fisher’s exact test was utilized. The normality of the distribution of quantitative variables was assessed using the non-parametric Kolmogorov–Smirnov test, along with the evaluation of skewness and kurtosis indices. A significance level of 0.05 was considered for all tests. The Paired-sample T test was performed to explore within-group (Pre– and post-values) differences and the Cohen's d was calculated to evaluate the effect size (To interpret the resulting number, can use this general guide developed by Cohen:

-  < 0.1 = trivial effect
- 0.1–0.3 = small effect
- 0.3–0.5 = moderate effect
-  > 0.5 = large difference effect) [36].

## Results

A total of 124 out of 126 participants completed the whole study and their results were analyzed. There were 2 participants who dropped out: Otago (1. low adherence (self-reported by participant)), Control (1. Unwillingness to continue cooperation (self-reported by participant)). There were no research-related injuries.

Average age was 63.33 (SD 2.4) years, BMI was 27.83 (SD 5.07), and number of fall was 0.75 (SD 1.04). 77.4% of participants were female, 66.9% of them had diploma and sub-diploma educational status, and 89.5% of them were married. Moreover, no significant differences were observed between-group for variables at baseline (Table 2).

**Table 2:** Participant demographic data

Variable	Mean ± SD				p-value
	CSQ group (n = 42)	Otago group (n = 41)	Control group (n = 41)	Total	
Age	63.67 ± 3.16	63.07 ± 2.01	63.24 ± 2.10	63.33 ± 2.48	0.537
BMI	27.42 ± 4.82	27.66 ± 4.73	28.43 ± 5.69	27.83 ± 5.07	0.643
Number of fall	0.67 ± 1.00	0.63 ± 0.79	0.95 ± 1.28	0.75 ± 1.04	0.323
Gender					
Male	10 (23.8%)	8 (19.5%)	10 (24.4%)	28 (22.6%)	0.846
Female	32 (76.2%)	33 (80.5%)	31 (75.6%)	96 (77.4%)	
Educational status					
Diploma and sub-diploma	24 (28.9%)	30 (36.1%)	29 (34.9%)	83 (66.9%)	0.246
Associate and above	18 (43.9%)	11 (26.8%)	12 (29.3%)	49 (33.1%)	
Marital status					
Married	36 (85.7%)	38 (92.7%)	37 (90.2%)	111 (89.5%)	0.575
Deceased spouse	6 (14.3%)	3 (3.7%)	4 (9.8%)	13 (10.5%)	

*BMI* body mass index; *SD* standard deviation; *CSQ* chair squat

Significance difference *p < 0.05

After the intervention, the SAFFE score significantly improved from 12.42 ± 1.768 to 10.21 ± 1.646 (p = 0.001) in the CSQ group, and from 12.65 ± 1.542 to 7.05 ± 1.071 (p = 0.001) in the Otago group but there was no significant improvement in the control group (from 12/09 ± 1/593 to 12/05 ± 1/413 (p = 0.599). It means that the average score of fear of falling in the CSQ and OEP intervention groups had decreased significantly, but in the control group, the mean score of fear of falling has not changed significantly from before to after the intervention. The improvement in the Otago group was significantly higher than that in the CSQ group (p = 0.001). There were significant differences (p = 0.001) among the three groups in the SAFFE score. According to the Cohen's d, the effect size of CSQ on the SAFFE score was large. The effect size of OEP on the SAFFE score was large too. But, OEP effect size was larger than CSQ on the SAFFE score. In the control group the effect size was small (Tables 3 and 5).

**Table 3:** The SAFFE score before and after the intervention (mean ± SD)

Group	Mean ± SD			Cohen's d	Post-hoc Tukey	
	Pre-intervention	Post-intervention	p-value		Comparing	p-value
SAFFE score						
CSQ (n = 42)	12.42 ± 1.768	10.21 ± 1.646	0.001*	1.293	Otago	0.001**
					Control	0.001**
Otago (n = 41)	12.65 ± 1.542	7.05 ± 1.071	0.001*	4.218	CSQ	0.001**
					Control	0.001**
Control (n = 41)	12/09 ± 1/593	12/05 ± 1/413	0.599	0.219	Otago	0.001**
					CSQ	0.001**
p-value	0.301	0.001**				

*SD* standard deviation; *CSQ* chair squat

*p value derived from paired-sample T test p < 0.016 (Bonferroni correction)

**p value derived from one-way ANOVA p < 0.05

After the intervention, the SF-36 score significantly improved from 43.18 ± 9.041 to 60.22 ± 4.902 (p = 0.001) in the CSQ group, and from 43.92 ± 9.890 to 63.08 ± 5.430 (p = 0.001) in the Otago group, and significantly decreased in the control group from 44.19 ± 8.588 to 42.44 ± 7.342 (p = 0.002). It means that the average score of quality of life improved significantly in the CSQ and OEP intervention groups, but in the control group, the mean score of quality of life decreased significantly from before to after the intervention. There was no significant difference between the Otago and the CSQ group (p = 0.079) but the differences between the Otago and the CSQ groups with the control group were significant (p = 0.001). According to the Cohen's d, the effect size of CSQ on the SF-36 score was large. The effect size of OEP on the SF-36 score was large too. But, CSQ effect size was larger than OEP on the SF-36 score. In the control group the effect size was small (Tables 4 and 5).

**Table 4:** The SF-36 score before and after the intervention (mean ± SD)

Group	Mean ± SD			Cohen's d	Post-hoc test	
	Pre-intervention	Post-intervention	*p-value		Comparing	p-value
SF-36 score						
CSQ (n = 42)	43.18 ± 9.041	60.22 ± 4.902	0.001	4.218	Otago	0.079
					Control	0.001**
Otago (n = 41)	43.92 ± 9.890	63.08 ± 5.430	0.001	2.401	CSQ	0.079
					Control	0.001**
Control (n = 41)	44.19 ± 8.588	42.44 ± 7.342	0.002	0.219	Otago	0.001**
					CSQ	0.001**
**p-value	0.872	0.001				

*SD* standard deviation; *CSQ* chair squat

*p value derived from Paired-sample T test

**p value derived from one -way ANAVA

**Table 5:** The effect sizes and confidence intervals for post hoc Tukey analysis

Group		SAFFE				SF-36			
(I) Group	(J) Group	Mean difference (effect size)	p-value	95% confidence interval		Mean difference (effect size)	p-value	95% confidence interval	
				Lower bound	Upper bound			Lower bound	Upper bound
CSQ (*n* = 42)	Otago	3.166*	0.001*	2.44	3.89	– 2.861	0.079	– 5.97	0.25
	Control	– 1.834*	0.001*	– 2.56	– 1.11	17.786*	0.001*	14.67	20.90
Otago (*n* = 41)	Squat	– 3.166*	0.001*	– 3.89	– 2.44	2.861	0.079	– 0.25	5.97
	Control	– 5.000*	0.001*	– 5.73	– 4.27	20.647*	0.001*	17.52	23.78
Control (*n* = 41)	Squat	1.834*	0.001*	1.11	2.56	– 17.786*	0.001*	– 20.90	– 14.67
	Otago	5.000*	0.001*	4.27	5.73	– 20.647*	0.001*	– 23.78	– 17.52

*The mean difference is significant at the 0.05 level

Before the intervention, there was no significant difference between the three groups in terms of SAFFE score (p = 0.301) and SF-36 score (p = 0.872) (Tables 3 and 4).

After the intervention, there was a significant difference between the three groups in terms of the SF-36 score (p = 0.001). Also, there was a significant difference between the three groups in terms of the SAFFE score (p = 0.001). According to the post hoc Tukey analysis, no significant difference was observed in the SF-36 score between the CSQ and OEP intervention groups (p = 0.079) but there was a significant difference in the SAFFE score between CSQ and OEP intervention groups (p = 0.001). Also, There was a significant difference in SF-36 score and SAFFE between the intervention and the control groups (p = 0.001) (Tables 3 and 4). In Table 5 effect size of post hoc Tukey analysis was summarized.

## Discussion

In this study the effect of Otago and chair squat exercises on the fear of falling and the quality of life of the older adults was compared. The main findings of the present study showed that after the intervention the average score of fear of falling in the CSQ and OEP intervention groups had decreased significantly, but in the control group, the mean score of fear of falling has not changed significantly from before to after the intervention. Also the average score of quality of life improved significantly in the CSQ and OEP intervention groups, but in the control group, the mean score of quality of life decreased significantly from before to after the intervention. These findings revealed that eight weeks of performing the OEP and CSQ at home improved QOL and decreased FOF in the older adults, so that the OEP effect on FOF reduction was greater. These findings seem to be the result of older adults’s physical activities. Moreover, the QOL decreased in the control group, which can be considered due to its well-being, role dysfunction due to physical health, and pain from QOL subscales.

Aging makes the older adults prone to falls and subsequently causes fear of engaging in activities, fear of falling and decrease in quality of life [37].

A recent quasi-experimental study concluded that balance exercises had a long-term impact on the improvement of balance and reduction of FOF in the older adults [38]. Also, a systematic review revealed that exercise activities were associated with a mild to moderate reduction in FOF [39]. Five weekly OEP training sessions with people aged 65–80 lead to an overall reduction in FOF [40]. Similarly, five-six 40–60-min sessions per week of OEP at home over eight weeks caused balance improvement and FOF reduction [41]. These previous results are consistent with those obtained in the present study, in which the OEP and CSQ had a beneficial effect on the reduction of FOF as physical activities and the OEP impact was greater. Against this, twelve weeks of OEP training had no effect on FOF in the older adults over the age of 65. [42]. The difference in the results of these two studies can be because of the difference in the training and implementation protocol of exercises and the evaluation criteria for fear of falling. As far as our knowledge allows, no study has been conducted to investigate the effect of CSQ on FOF in the older adults.

A cross-sectional study concluded that QOL and physical activity are significantly correlated [14]. Also, a clinical trial study supported that performing progressive exercises at home enhanced the QOL of community older adults over 60 (88% female) [43]. Performance of three 60-min sessions per week for eight week home-based OEP increased QOL in person over 60 in Iran, Arak [29]. These conclusions were consistent with the present study results. The OEP and CSQ had a positive impact on the improvement of QOL. On the contrary, 50 min, three times per week for 8 weeks of OEP performance (2 group and 22 home programs) did not significantly improve QOL in stroke patients aged over 65 [44]. Among the reasons for the difference in the results of this study with the current study, we can mention that our measurement scales were different. They used EQ-5D questionnaire to assay the QOL but we used SF-36 questionnaire and also in this study, the participants were monitored and evaluated at the end of every week (after every three sessions), while in our study, they were monitored and evaluated before the beginning and at the end of each session. Also, a systematic review and meta-analysis concluded that OEP is not significantly effective in improving the quality of life of older adults with frailty and pre-frailty [45]. In our study, the participants did not have frailty and disability and the method was different. No study had investigated the effect of CSQ on the older adults QOL as we searched.

There is an inverse relationship between FOF and QOL, so reducing FOF leads to an increase in QOL [46] and people who have a falling history have a lower quality of life [6]. Also, QOL is related to the intensity of pain perceived by the older adults [47].

One of the current study's highlights was performing exercises at home, which encouraged participants to exercise at home due to their conditions and transportation limits.

The results of this study revealed that the implementation of regular and organized exercise programs at home led to a significant reduction in the fear of falling in the older adults and increasing physical activities at home can significantly improve the quality of life of them [48]. In addition, this method can support the elderly who exercise at home to easily stick to their plans. This method has not only played an important role in improving the quality of life of the elderly, but also provided effective solutions to reduce the fear of falling in this group of people [43, 49]. Therefore, it is recommended that these exercise programs be considered as an essential part of health care and supportive plan for the older adults.

## Conclusion

We can conclude that doing home-based OEP for eight weeks had better effects on reducing FOF, and doing home-based OEP and CSQ for eight weeks had beneficial effects on improving QOL among the older adults. Less complicated home-based exercises such as OEP and CSQ can be suggested to the older adults as cost-effective exercises to be done regularly to help them avoid complications of old age like fear of falling and low quality of life.

### Limitation

Participants engaged in exercises independently at home for 8 weeks. The lack of direct supervision regarding the execution of the exercises and accurate adherence to the protocols can be considered a limitation of this study. Another limitation of the study is that intention to treat analysis was not performed.

## Data Availability

The data that support the findings of this study are available from the corresponding author upon reasonable request.
